# Synthesis of sharply thermo and PH responsive PMA-b-PNIPAM-b-PEG-b-PNIPAM-b-PMA by RAFT radical polymerization and its schizophrenic micellization in aqueous solutions

**DOI:** 10.1080/15685551.2017.1314654

**Published:** 2017-05-08

**Authors:** Lida Ahmadkhani, Mojtaba Abbasian, Abolfazl Akbarzadeh

**Affiliations:** ^a^ Department of Chemistry, Payame Noor University (PNU), Tehran, Iran; ^b^ Department of Basic Science, Payame Noor University (PNU), Tehran, Iran; ^c^ Faculty of Advanced Medical Science, Department of Medical Nanotechnology, Tabriz University of Medical Sciences, Tabriz, Iran; ^d^ Drugs Applied Research Center, Tabriz University of Medical Sciences, Tabriz, Iran

**Keywords:** Pentablock terpolymer, RAFT polymerization, stimuli-responsive, core–shell–corona micelles, temperature, pH-responsive, PNIPAM: Poly(N-isopropylacrylamide), PAAm: Polyacrylamide, PAA: Poly(acrylic acid), PMA: Poly(methacrylic acid), P4VP: Poly(4-vinylpyridine), LCST: Lower Critical Solution Temperature, AA: Acrylic Acid, MAA: Methacrylic Acid, NIPAM: N-isopropylacrylamide, CRP: Controlled radical polymerization, RAFT: Reversible Addition–Fragmentation chain Transfer, NMP: Nitroxide Mediated Polymerizations, ATRP: Atom Transfer Polymerizations, PEG: Poly(ethylene glycol), TEM: Transmission Electron Microscopy, SEM: Scanning Electron Microscopy, GPC: Gel Permeation Chromatography, AIBN: Azobisisobutyronitrile, THF: Tetrahydrofuran, DMF: Dimethylformamide, DCC: Dicyclohexylcarbodiimide, DMAP: 4-dimethylaminopyridine, CTA: Chain Transfer Agent, DCM: Dichloromethane, DCU: Dicyclohexylurea, CMC: Critical Micelle Concentration, FT-IR: Fourier Transform Infrared spectroscopy, H NMR: Nuclear Magnetic Resonance spectroscopy, TMS: Tetramethylsilane, UV–vis: Ultraviolet-visible, PDI: polydispersity index

## Abstract

Sharply thermo- and pH-responsive pentablock terpolymer with a core-shell-corona structure was prepared by RAFT polymerization of N-isopropylacrylamide and methacrylic acid monomers using PEG-based benzoate-type of RAFT agent. The PEG-based RAFT agent could be easily synthesized by dihydroxyl-capped PEG with 4-cyano-4-(thiobenzoyl) sulfanylpentanoic acids, using esterification reaction. This pentablock terpolymer was characterized by ^1^H NMR, FT-IR, and GPC. The PDI was obtained by GPC, indicating that the molecular weight distribution was narrow and the polymerization was well controlled. The thermo- and pH-responsive micellization of the pentablock terpolymer in aqueous solution was investigated using ﬂuorescence spectroscopy technique, UV–vis transmittance, and TEM. The LCST of pentablock terpolymer increased (over 50 °C) compared to the NIPAM homopolymer (~32 °C), due to the incorporation of the hydrophilic PEG and PMA blocks in pentablock terpolymer (PNIPAM block as the core, PEG the block and the hydrophilic PMA block as the shell and the corona). Also, pH-dependent phase transition behavior shows at a pH value of about ~5.8, according to pKa of MAA. Thus, in acidic solution at room temperature, the pentablock terpolymer self-assembled to form core–shell–corona micelles, with the hydrophobic PMA block as the core, the PNIPAM block and the hydrophilic PEG block as the shell and the corona, respectively.

## Introduction

In recent decades, dual responsive copolymers in aqueous solutions have attracted considerable attention, because of its application in gene and drug delivery, polymeric surfactants for stabilization of colloid dispersions and separations. Stimuli-responsive polymers are classified into two groups: (1) polymers responsive to the body’s internal stimuli such as pH-, temperature-, ion-, redox-, enzyme-, and biomolecule-sensitive polymers and (2) polymers responsive to external stimuli such as polymers sensitive to CO_2_, temperature, magnetic fields, light and electrics fields [1–9].

Responsive polymers show reversible transitions in properties. These polymers are able to undergo a conformational change of phase transition upon the application of an external stimulus such as a change in solution pH, electrolyte concentration or temperature [10–12]. At the macromolecular level, polymer chains can be changed in several ways, such as changes in hydrophilic-to-hydrophobic equilibrium, degradation, conformation, bond cleavage, and solubility and these properties lead to detectable behavioral changes to self-assembled structures [13]. The most important systems, also from a chemical and materials science point of view are those sensitive to pH or temperature.

Most studies were focused on smart biocompatible synthetic polymers, such as Poly(N-isopropylacrylamide) (PNIPAM), poly(4-vinylpyridine) (P4VP), polyacrylamide (PAAm), poly(acrylic acid) (PAA), poly(methacrylic acid) (PMA), which, are sensitive to temperature and pH [14–17]. As an important component, PNIPAM and PAA have been widely used to compose block copolymers and hydrogels [18] that are thermo or pH sensitive. Recently, copolymers composed of PNIPAM blocks have gained much interest in biomedical due to the temperature sensitivity of the PNIPAM block [19,20].

An important feature of PNIPAM is the critical solution temperature that exhibits a lower critical solution temperature (LCST) in aqueous solution around 32 °C [21–23]. The LCST is ascribed to a change in the hydrophilic/hydrophobic balance of the polymer chains. At low temperatures of LCST, strong H-bonding interactions between polar groups and water lead to good solubility of the polymer, which is opposed by the hydration of a polar groups [24–26]. It has been shown that the LCST depends on the concentration, length of the chain [27] and polymer building (linear or branched) [28]. The LCST of such thermo sensitive polymers can be adjusted to an optimal temperature range by copolymerization with a hydrophilic copolymer (which increases the LCST) or a more hydrophobic copolymer (which lowers the LCST) [29].

Block copolymers and hydrogels involving of poly(acrylic acid) and poly(N-isopropyl-acrylamide) are of great attention for a diversity of aims [30–35], in addition to PEG as a usual hydrophilic polymers, has numerous biomedical and pharmaceutical applications because of its specific properties such as non toxicity, biocompatibility, biodegradability, and resistance to recognition by the immune system, therefore, polymeric micelles consisting of PEG have also been attracted. Poly(acrylic acid) is pH-responsive polymers, which, can answer back to pH changes to change their morphology and conformation in the environments due to the protonation-deprotonation equilibrium of the carboxyl group in aqueous solution [36,37]. The carboxylic groups can receive protons at pH < pKa and release protons at pH > pKa; as a result, the balance between electrostatic repulsion forces and hydrogen bonds makes the change of hydrophobic/hydrophilic features of PAA at different pH [38–40].

It has been found that some of the stimuli-responsive polymeric micelles of poly (N-isopropylacrylamide) and poly (acrylic acid) blocks show schizophrenic behavior. Here, the schizophrenic behavior means that some AB-type diblock copolymers can create two different micelle structures, for example, the A-core/B-corona arrangement and B-core/A-corona arrangement, and the two structures can be reversibly converted to each other by some stimuli [41–44].

Easily available carboxylic acid monomers, such as acrylic acid (AA) or methacrylic acid (MAA), have been copolymerized with N-isopropylacrylamide using traditional free radical polymerization methods, to form random copolymers with both thermo and pH-responsive properties [45,46].

To attain the copolymers having well-define structure and morphologies, controlled radical polymerization (CRP) techniques is a better method, which can yield well-deﬁned high molecular weight and narrow molecular weight distributions.

The growth of controlled/living polymerizations has allowed synthetic polymer chemists to proposal complex polymeric buildings with high accuracy [47]. Reversible addition–fragmentation chain transfer (RAFT) polymerizations [48,49], atom transfer polymerizations (ATRP) [50–52] and nitroxide mediated polymerizations (NMP) [53] yield well-defined macromolecules with low polydispersity [54]. Among these basic techniques, RAFT demonstrates the most useful and facile method, since it uses a diversity of chain transfer agents [55] allowing a broad range of monomers to be polymerized without the use of transition metal catalysts. RAFT polymerization is more facile and strong method for the synthesis of various types of macromolecules and water-soluble polymers with favorite molecular design and functionality, as it can be applied to almost any type of monomer without the necessity of employing protecting functional groups. RAFT polymerization permits the use of acidic monomers and also the use of polar solvents such as ethanol [56].

In the present study, a combination of PNIPAM, PMA, and PEG in an ABCBA pentablock terpolymer, PMA-b-PNIPAM-b-PEG-b-PNIPAM-b-PMA was synthesized using RAFT polymerization to yield controlled and narrow molecular weight distributions. The pH- and thermo-responsive micellization manners were studied using various conditions. The structure and dually responsive properties of the pentablock terpolymers were assayed by FT-IR, ^1^H NMR, UV–vis, scanning electron microscopy (SEM), transmission electron microscopy (TEM), gel permeation chromatography (GPC).Containing pH-responsive PMA and thermo-responsive PNIPAM, ABCBA-type hydrophilic pentablock terpolymers molecularly dissolved in aqueous solution at neutral pH and room temperature, but macromolecular self-assembled into PMA-core micelles at acidic pH and room temperature, and PNIPAM-core micelles at neutral pH and high-temperature.

## Experimental

### Material and methods

Methacrylic acid (Merck, >99%) was distilled under vacuum before use. Azobis-isobutyronitrile (AIBN) is an initiator, which, was recrystallized from methanol former to use. Tetrahydrofuran (THF) (Scharlau, >99%), *n*-hexane (Merck, >99%), dimethylformamide (DMF) (Aldrich, 99%), dichloromethane (DCM) (Chem-Lab, >99.8%), chloroform (Mojallali, >99%), ethanol (Merck, 99.9%), methanol (Merck, 99.9%) were used as-purchased without further puriﬁcation. N-isopropyl-acrylamide (NIPAM) (Aldrich) was recrystallized from *n*-hexane. Polyethylene glycol (PEG), biological grade, with molecular weight (*M*_*w*_) of 4000 was supplied by the Sigma-Aldrich and used as received. 4,4′-azobis(4-cyanopentanoic acid) (75% Aldrich), bromobenzene (Aldrich, 99%), carbon disulfide (CS_2_, 99.9%), ethyl acetate, iodine (Aldrich, 99%) and magnesium sulphate (Aldrich, 98%), dicyclohexylcarbodiimide (DCC) and 4-dimethylaminopyridine (DMAP), were used as-purchased without further puriﬁcation.

### Characterization and measurements

FT-IR characterization was recorded using Shimadzu FT-IR-8101M spectrometer. All the spectrum were measured at a resolution 4 cm^−1^ and scanned from 4000 to 400 cm^−1^. The samples were prepared by grinding the dry powders with KBr pellets and compressing the mixture to form disks. ^1^H NMR of synthesized polymers were performed on FT-NMR (400 MHz) spectrometer (Bruker, Ettlingen, Germany). CDCl_3_ was used as the solvent at 25 °C, and chemical shifts were reported in ppm units with tetramethylsilane (TMS) as an internal standard. The chemical structures of products from each synthesis step were characterized by ^1^H NMR with CDCl_3_ as solvents. The molecular weight and its distribution (*M*_*w*_/*M*_*n*_) of the triblock copolymer and pentablock terpolymer were determined by gel permeation chromatography (GPC) (Ventura, CA, U.S.A.), equipped with a series of styragel columns. Tetrahydrofuran (THF) was used as an eluent at a ﬂow rate of 1 mL/min and pump pressure maintained was 3 mPa at 25 °C. Polystyrenes with narrowly distributed molecular weights were used as standards for calibration. Ultraviolet-visible spectroscopy was performed using 1650 PC UV–vis spectrophotometer (Shimadzu, Kyoto, Japan). The pH-sensitivity, the lower critical solution temperature (LCST) and critical micelle concentration (CMC) were determined by UV–vis spectroscopy at a wave length of 495 nm. The temperature of the sample cells was increased from 25 to 65 °C. The morphologies of pentablock terpolymer were obtained by transmission electron microscopy (TEM; PHILIPS CM10 TEM EPSON HP8300) and scanning electron microscopy (SEM; Cam Scan MV 2300). The samples were coated with Au before observation.

### Synthesizing procedures

A multi-responsive hydrophilic ABCBA-type pentablock terpolymer, consisting of poly(ethylene glycol), poly(N-isopropylacrylamide), and poly(methacrylic acid), PMA-b-PNIPAM-b-PEG-b-PNIPAM-b-PMA, were synthesized according to the following four steps: (1) preparation of RAFT agent (CTA); (2) preparation of macromolecular chain transfer agent, CTA–PEG–CTA; (3) synthesis of triblock copolymer (PNIPAM-b-PEG-b-PNIPAM); (4) synthesis of pentablock terpolymer (PMA-b-PNIPAM-b-PEG-b-PNIPAM-b-PMA) to achieve dual-responsive hydrophilic polymers.

### Synthesis of 4-cyano-4-((thiobenzoyl)sulfanyl)pentanoic acid a RAFT agent

**Scheme 1.**
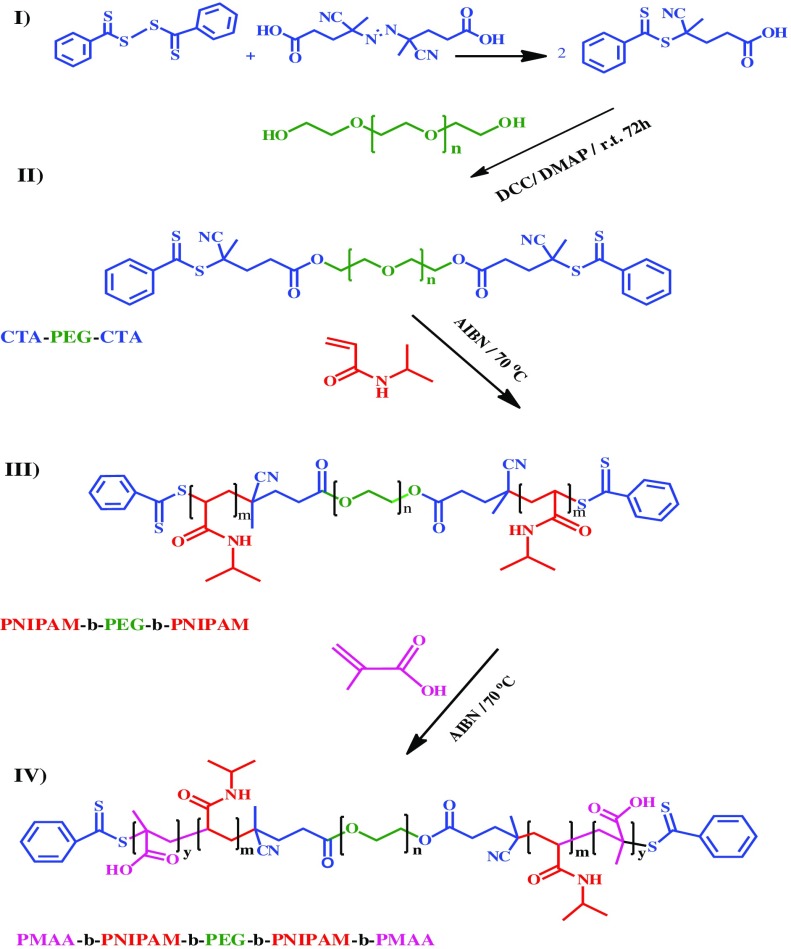
Synthesizing procedures of pentablock terpolymer of PMA-b-PNIPAM-b-PEG-b-PNIPAM-b-PMA.

Bis(thiobenzoyl) disulﬁde was prepared according to the reported procedure by Le et al. [57]. The target compound 4-cyano-4-((thiobenzoyl)sulfanyl)pentanoic acid was synthesized by heating the mixture of diphenyl dithioperoxy anhydride (1.62 g), and 4,4′-azobis(4-cyanopentanoic acid) (1.62 g) in 60 mL of ethyl acetate in 85 °C for 18 h, while purging with nitrogen. After eliminating the solvent using a rotary evaporator, the crude product that was obtained was subjected to column chromatography using a mixture of ethyl acetate and *n*-hexane with a ratio of 3/2, to yield an oily red compound (2.23 g, 69%) (Scheme 1).

### Synthesis of PEG macro-RAFT agent (CTA–PEG–CTA)

The synthesis of the CTA–PEG–CTA, chain transfer agent was carried out by the reaction of dihydroxyl-capped PEG with 4-cyano-4-((thiobenzoyl)sulfanyl)pentanoic acid, chain transfer agent (CTA) with the assistance of 4-dimethylaminopyridine and dicyclohexyl-carbodiimide in methylene dichloride. Brieﬂy, in a 250 mL one-neck round-bottom ﬂask equipped with a magnetic stirring bar, CTA (0.81 g, 3.0 mmol) and HO–PEG–OH (4 g, 1.0 mmol) were dissolved in 70 mL of DCM. After the solution was homogenized by stirring, the ﬂask was placed in an ice bath. Then, DCC (0.50 g, 2.4 mmol) and DMAP (0.03 g, 0.24 mmol) were added. After 30 min of stirring at 0 °C, the reaction temperature is raised to room temperature, then, stirred for 2 days. The precipitated dicyclohexylurea (DCU) was removed by ﬁltration. The ﬁltrate was concentrated by rotary evaporator and precipitated into excess diethyl ether twice to remove the unreacted CTA, PEG macro-CTA with light yellow color was obtained by ﬁltering and drying under vacuum at room temperature for 48 h (3.4 g, 85%) (Scheme 1).

### Synthesis of PNIPAM-b-PEG-b-PNIPAM triblock copolymers by RAFT polymerization

After the successful synthesis of CTA–PEG–CTA, it was employed in the RAFT polymerization of NIPAM, as depicted in Scheme 1. Briefly, CTA–PEG–CTA (0.7 g, 0.17 mmol), NIPAM (1.32 g, 24.7 mmol), and AIBN (7.18 mg, 0.044 mmol) were dissolved in THF (3 mL) inside a round-bottomed flask. After oxygen was removed by purging argon, the sealed flask was immersed in a temperature controlled oil bath kept at 65 °C and stirred for 24 h. After polymerization, the reaction mixture was poured in to an excess amount of diethyl ether. The product was collected by ﬁltration and puriﬁed twice by dissolution/precipitation with methylene chloride/diethyl ether and then dried in a vacuum for 24 h (1.3 g, 64%) (Scheme 1).

### Synthesis of PMA-b-PNIPAM-b-PEG-b-PNIPAM-b-PMA pentablock terpolymer by RAFT polymerization

The procedure used for the formation of pentablock terpolymer was similar to those used for the synthesis of the triblock copolymer. Synthesisof pentablock terpolymer was carried out by the chain extension of PNIPAM-b-PEG-b-PNIPAM macro-RAFT agent with methacrylic acid as the second monomer via RAFT technique. A typical procedure was as follows: Triblock copolymer (0.5 g), methacrylic acid (1.06 g, 14.7 mmol) and AIBN (2.4 mg, 1.45 × 10^−5^ mol) in 3 ml 1,4-dioxane were added to a polymerization flask. After oxygen was removed by purging argon, the sealed flask was placed in an oil bath at 65 °C. After the polymerization was stopped by cooling to 25 °C, the polymerization tube was opened to air. The mixture was diluted with 4 ml of methanol, and the polymer was precipitated with diethyl ether, then, filtered and dried under vacuum at 25 °C for 24 h (1.32 g, 84%) (Scheme 1).

### Self-assembly of pentablock terpolymer and CMC determination

Critical micelle concentration (CMC) of the pentablock terpolymers in aqueous solutions at 25 °C were estimated by ﬂuorescence probe method using pyrene as a ﬂuorescence probe. Pyrene was dissolved in acetone to make a concentration of 3 × 10^−6^ mol/L and then diluted by adding 10 mL different concentration of pentablock terpolymer aqueous solution. After evaporation of acetone, the mixed solutions were equilibrated at room temperature for 24 h before measurement. Excitation was carried at 338 nm.

## Results and discussion

### Synthesis and characterization of PMA-b-PNIPAM-b-PEG-b-PNIPAM-b-PMA pentablock terpolymer

The pentablock terpolymer was synthesized using the following four steps as shown in Scheme 1. ^1^H NMR, GPC and FT-IR analysis were employed to characterize the prepared polymer.

### First step: synthesis and characterization of 4-cyanopentanoic acid dithiobenzoate RAFT agent

**Figure 1.**
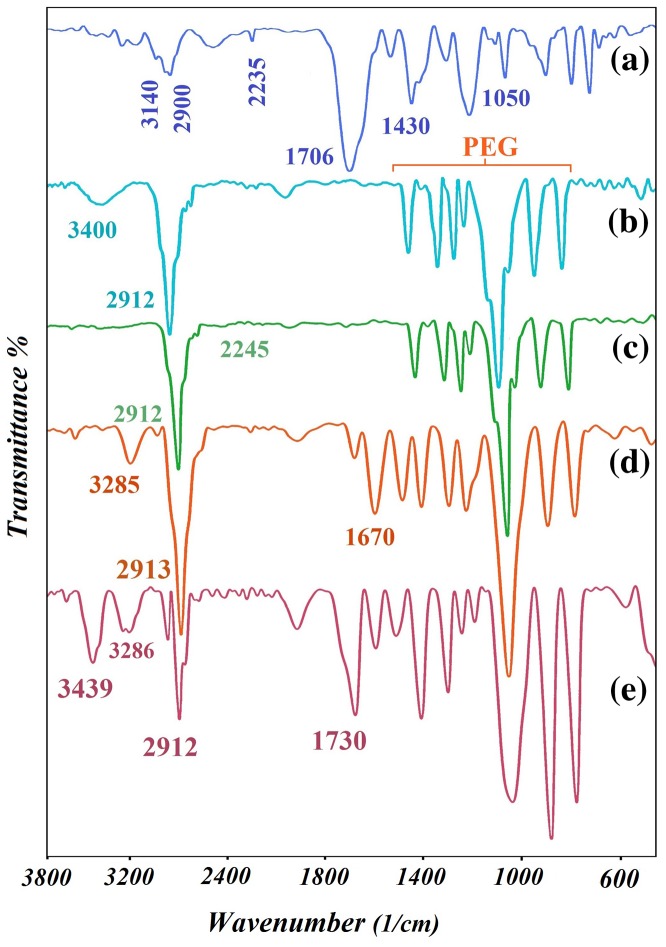
FT-IR of (a) 4-cyano-4-((thiobenzoyl)sulfanyl)pentanoic acid (b) PEG (c) CTA–PEG–CTA (d) triblock copolymer of PNIPAM-b-PEG-b-PNIPAM (e) pentablock terpolymer PMA-b-PNIPAM-b-PEG-PNIPAM-b-PMA.

4-cyanopentanoic acid dithiobenzoate RAFT agent synthesized and was conﬁrmed by FT-IR spectroscopy and ^1^H NMR. The FT-IR spectrum of 4-cyanopentanoic acid dithiobenzoate RAFT agent (CTA) is shown in Figure 1(a). From the FT-IR spectrum shown the absorption peak at ~2900–3140 cm^−1^ is related to the stretching vibration mode of C–H bonds aliphatic and aromatic and peaks at 2235 cm^−1^ ascribed to the stretching vibrations of –C≡N bonds, a strong band at 1706 cm^−1^ is dedicated to C=O stretch, absorption at 1430, 1050 and 576 cm^−1^ is due to C=C aromatic, C=S and C–S stretch, respectively. The peak observed in the area 3400 cm^−1^ related to stretching vibration of the hydroxyl group (OH).

**Figure 2.**
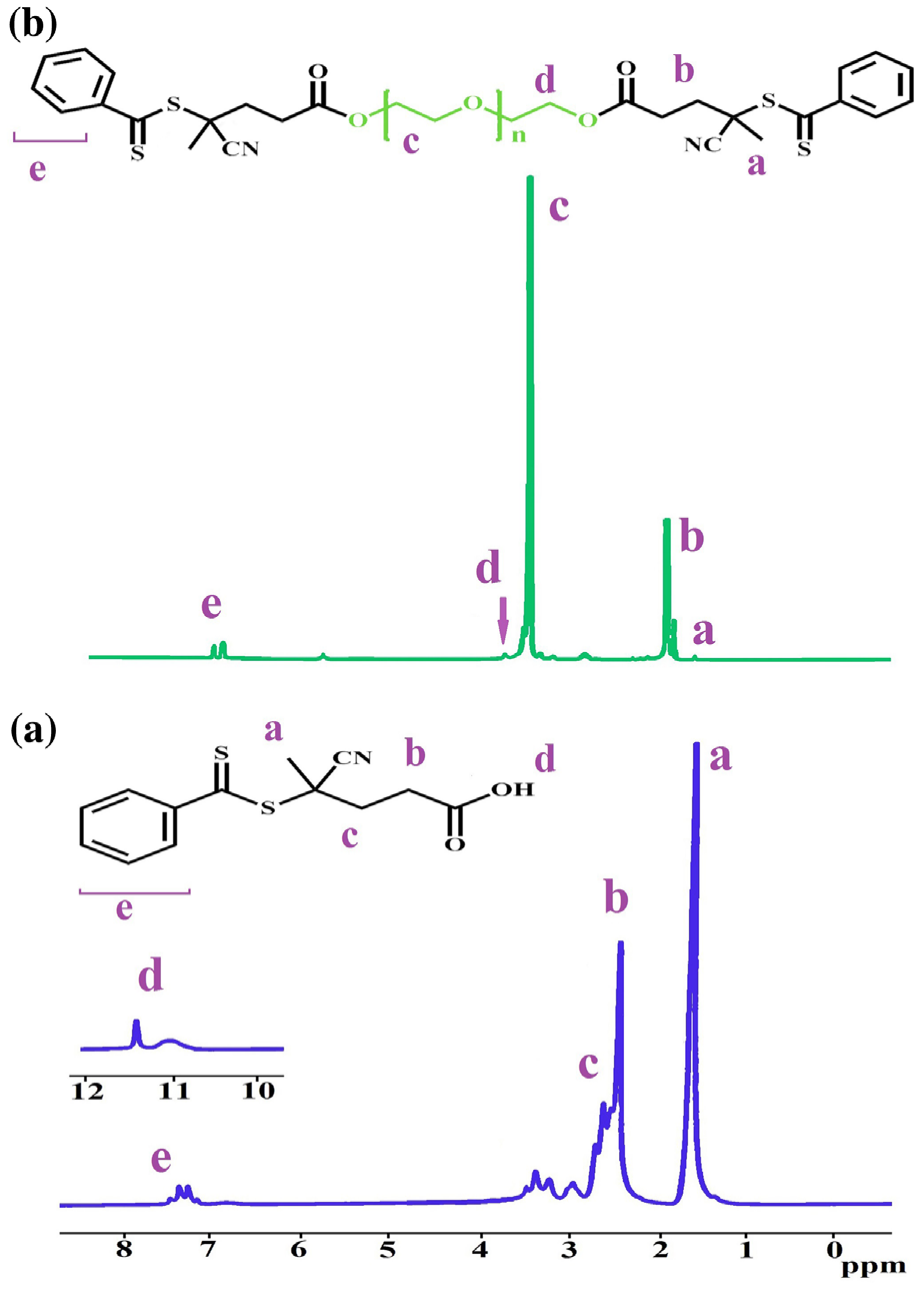
^1^H NMR spectrum of (a) 4-cyanopentanoic acid dithiobenzoate (b) CTA–PEG–CTA macro RAFT agent.

Nuclear magnetic resonance spectroscopy (^1^H NMR) study has been known as a powerful device for the characterization of organic compounds because of its facility, rapidity, and sensibility. The ^1^H NMR spectrum of 4-cyanopentanoic acid dithiobenzoate is demonstrated in Figure 2(a). The resonance of 4-cyanopentanoic acid dithiobenzoate is *δ* = 7.2–7.4 ppm (peak ‘e’) for the phenyl group, *δ* = 11.4 ppm (peak ‘d’) for carboxyl group and *δ* = 2.3–2.6 ppm (peak ‘b, c’) for the methylene and *δ* = 1.65 ppm (peak ‘a’) for the methyl groups of cyanopentanoic acid fragment. This ^1^H NMR spectrum assignment proves that the CTA had been successfully synthesis.

### Second step: synthesized and characterization of the CTA–PEG–CTA macro-RAFT agent

PEG-based macro-RAFT agent, CTA–PEG–CTA is prepared via the esteriﬁcation of the terminal dihydroxyl group of HO–PEG–OH with the terminal carboxyl group of RAFT agent. To confirm the structure, the FT-IR spectrum of HO–PEG–OH (b) and CTA–PEG–CTA (c) are shown in Figure 1. Two strong absorption peaks at 2912 and 1114 cm^−1^ are PEG characteristic modes attributable to the C–H and C–O stretching vibration, and the absorption bands at 1467 and 1345 cm^−1^ belong to C–H bending modes. As observed in Figure 1(c), the absorption for dithiocarbonate units at 1060 cm^−1^ (C=S) was not observed due to the overlap with the strong absorption of PEG between 960 and 1200 cm^−1^. On the other hand, the stretching vibration peak of –OH in CTA–PEG–CTA has been disappeared at the band 3400 cm^−1^, which shows the progress of the reaction, also, the stretching vibration peak of C≡N in CTA–PEG–CTA has appeared at the band 2245 cm^−1^.

^1^H NMR spectrum is further employed to verify the chemical structure of CTA–PEG–CTA, as shown in Figure 2. Figure 2(a) and (b) show the ^1^H NMR spectrum of RAFT agent and the as-prepared CTA–PEG–CTA macro-RAFT agent. Some new peaks are apparent in the ^1^H NMR spectrum of the macro-RAFT agent, compared with that of CTA; the peak at *δ* = 3.8 ppm (peak ‘d’), which corresponds to the proton of –CH_2_–O–(C=O), and the signal at *δ* = 3.52 ppm (peak ‘c’) which is assigned to the proton in the methylene of the PEG chain. The peak at *δ* = 1.8 ppm (peak ‘b’) corresponds to methylene of CTA. The new peaks prove that progress reaction was successful.

### Third step: RAFT polymerization of NIPAM using CTA–PEG–CTA as the RAFT agent

**Figure 3.**
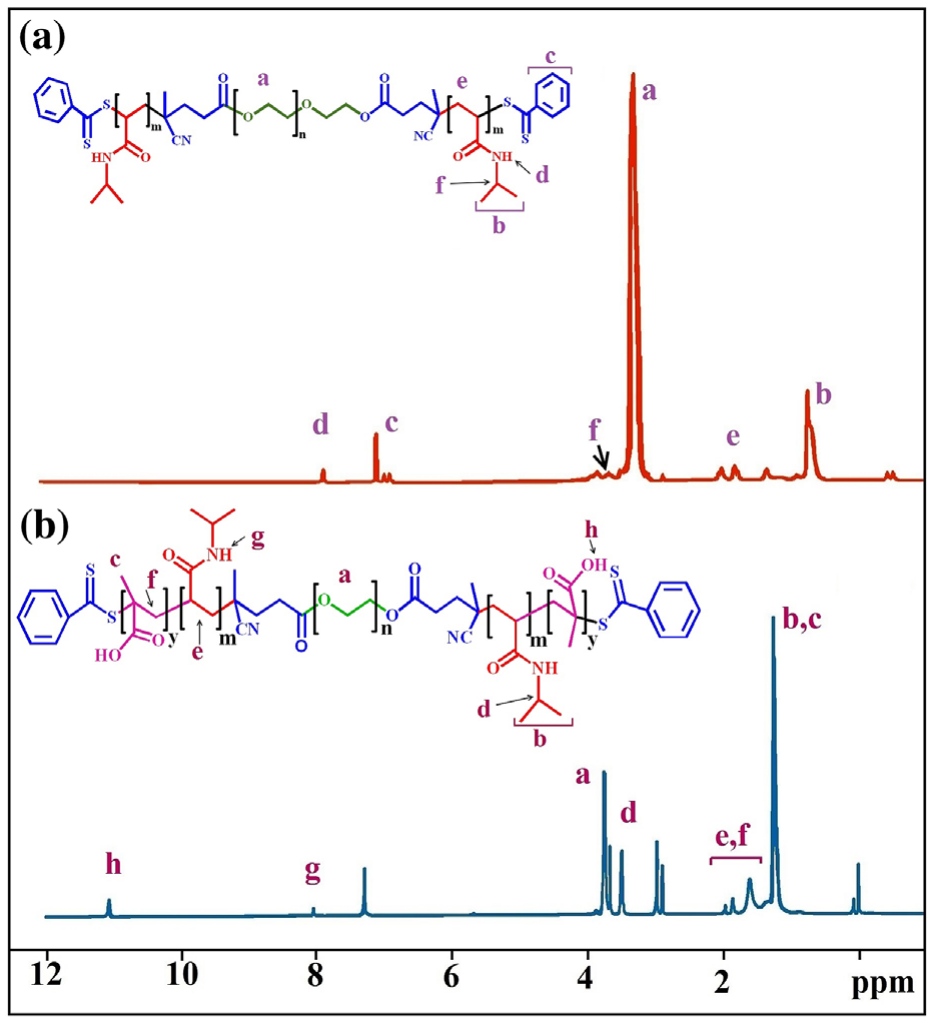
^1^H NMR spectrum of (a) triblock copolymer of PNIPAM-b-PEG-b-PNIPAM (b) pentablock terpolymer PMA-b-PNIPAM-b-PEG-PNIPAM-b-PMA.

**Figure 4.**
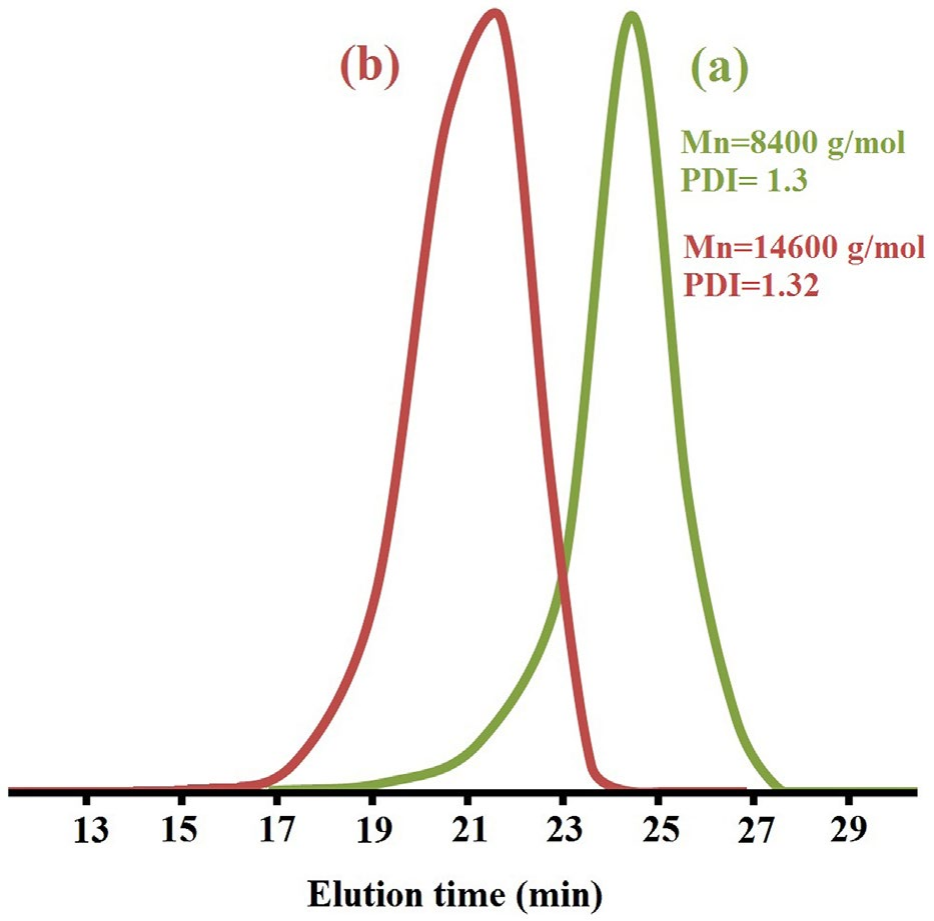
GPC traces of (a) PNIPAM-b-PEG-b-PNIPAM triblock copolymer, (b) PMA-b-PNIPAM-b-PEG-b-PNIPAM-b-PMA.

PNIPAM-b-PEG-b-PNIPAM triblock copolymers are then synthesized by RAFT polymerization of N-isopropylacrylamide (NIPAM) using CTA–PEG–CTA as macro-RAFT chain transfer reagent. The composition of the triblock copolymer was determined by ^1^H NMR, FT-IR and GPC analysis (Figures 1(d), 3(a) and 4).

**Figure 5.**
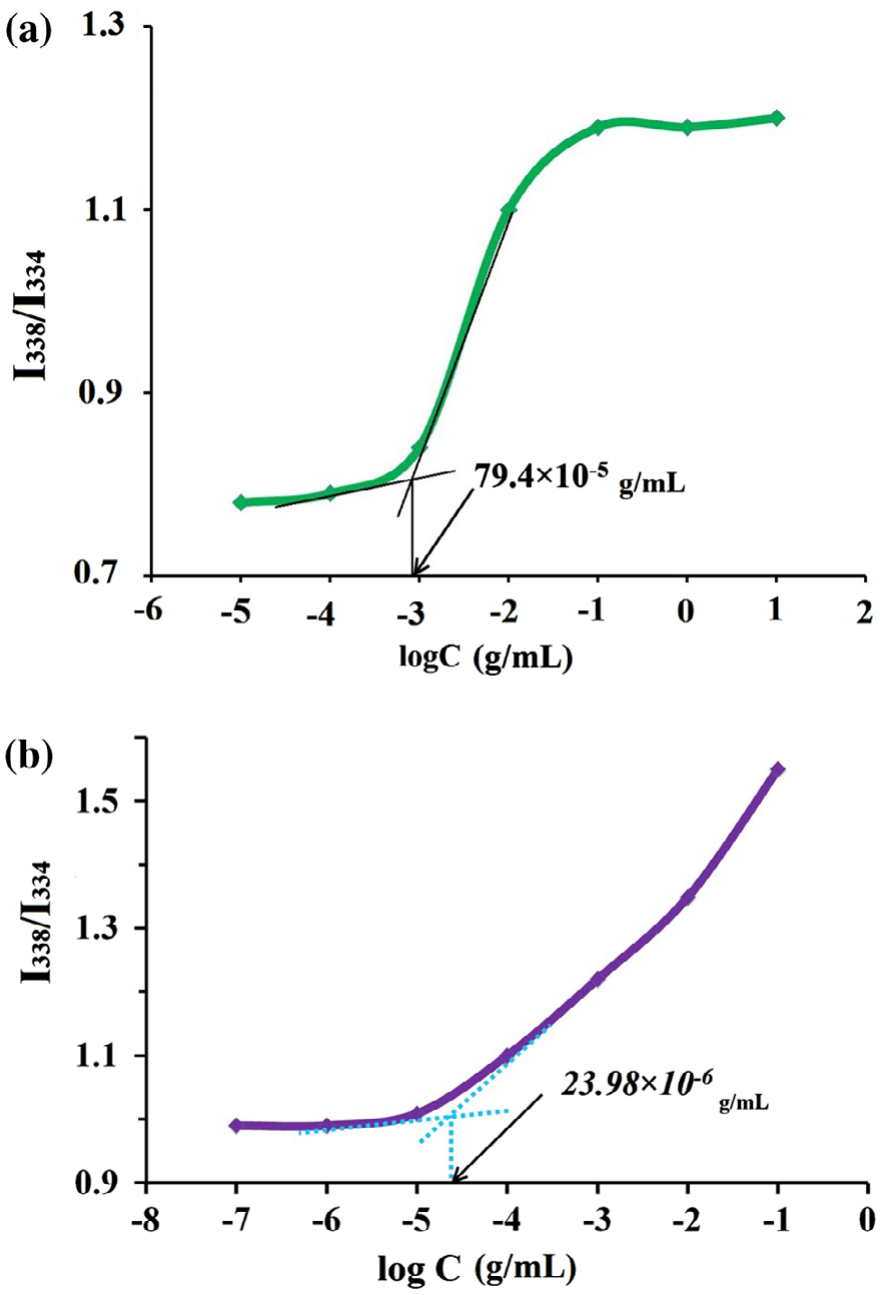
The fluorescence intensity *I*_338_/*I*_334_ of pentablock terpolymer solutions (a) 60 °C (b) pH 4.8.

**Figure 6.**
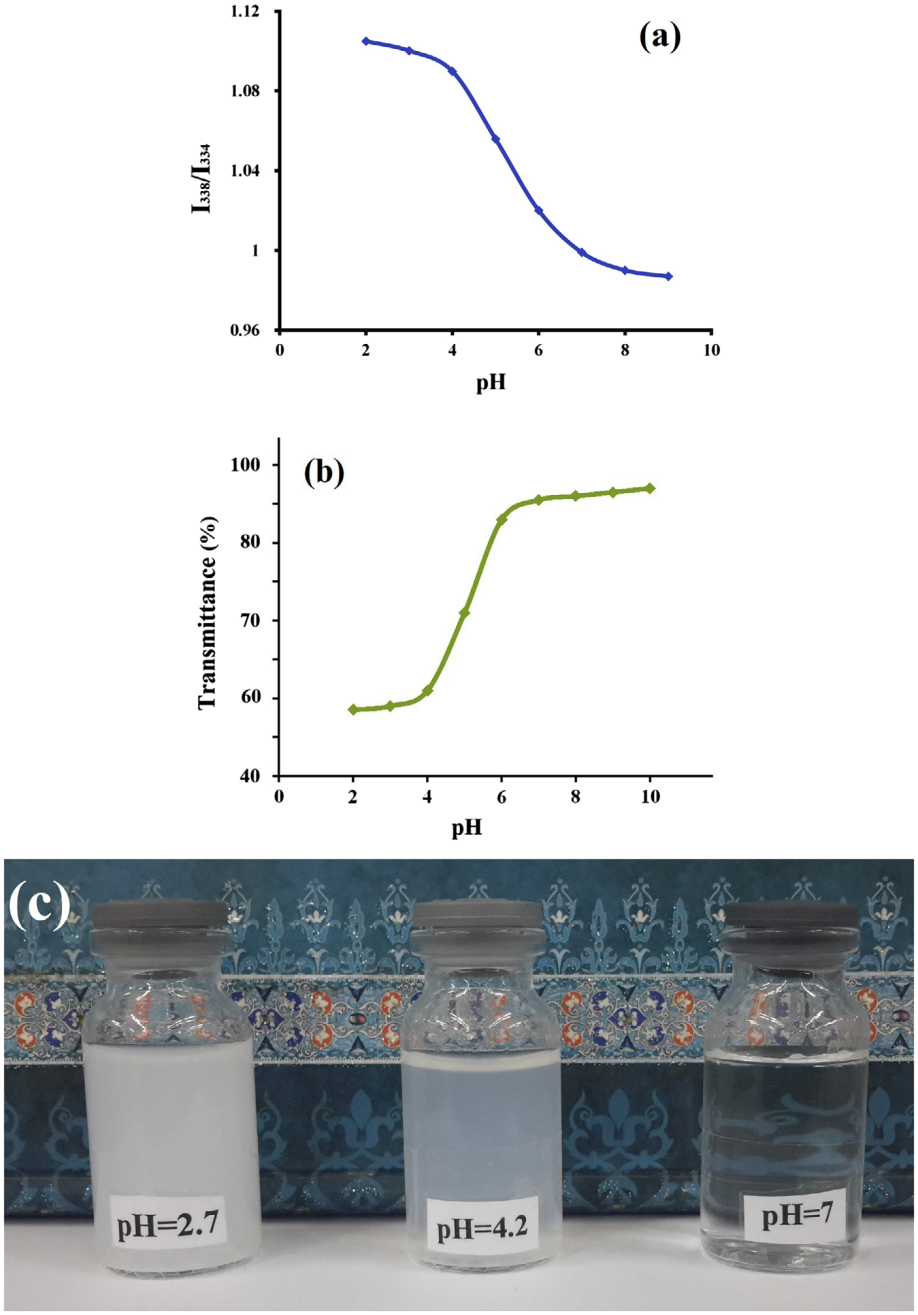
(a) Fluorescence intensity ratios *I*_338_/*I*_334_ from pyrene excitation spectrum and (b) UV–vis transmittance at 495 nm of pentablock terpolymer micelle as a function of pH values (*C* = 0.2 mg L^−1^) (c) Photo images of pentablock terpolymer at different pH.

**Figure 7.**
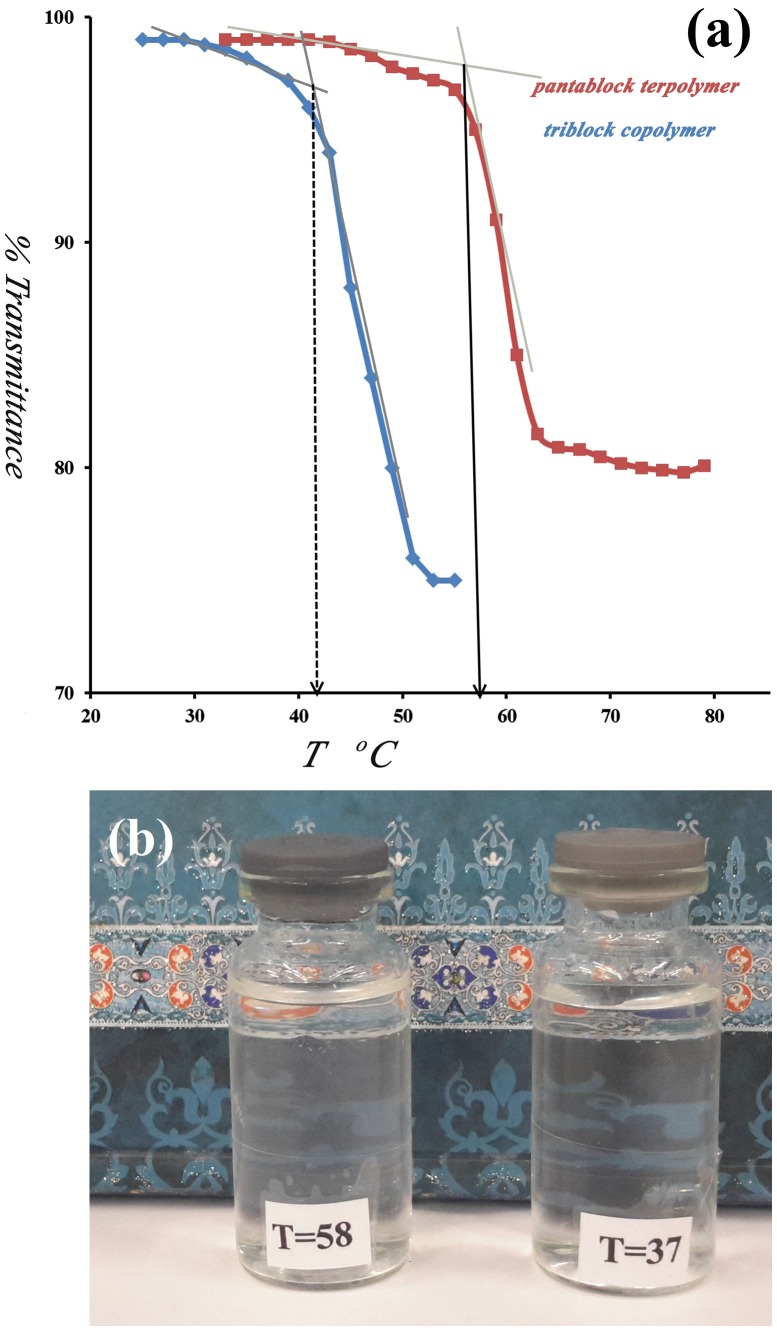
(a) Temperature dependence of the light transmittance of triblock copolymer and pentablock terpolymer solutions (*C* = 0.2 mg/L, pH 7), (b) Photo images of pentablock terpolymer at two different temperatures.

**Figure 8.**
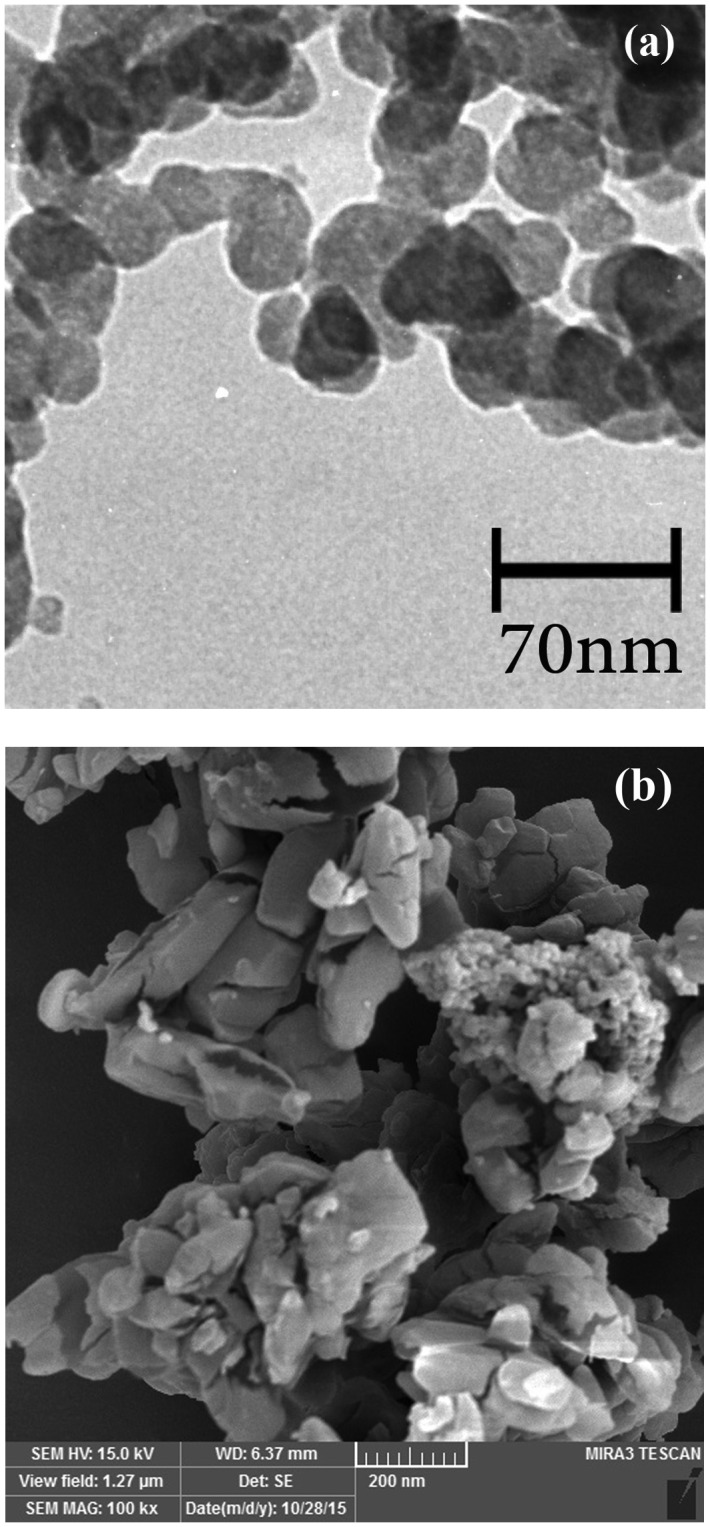
(a) Transmission electron microscopy (TEM) image of pentablock terpolymer of in pH 5 and (b) SEM image of pentablock terpolymer in the solid state.

Figure 1(d) shows FT-IR spectrum of PNIPAM-b-PEG-b-PNIPAM triblock copolymer. As exhibited in the FTIR spectrum, the absorbance of amide carbonyl groups in triblock copolymer occurred at 1670 cm^−1^ and the bending frequency of the amide N–H appeared at 1550 cm^−1^. The bands at 3285 cm^−1^ assigned to the stretching vibration of N–H as shown in FT-IR spectrum (Figure 1(d)), that, the monomers are successfully polymerized by RAFT.

^1^H NMR characterization of the resulting triblock copolymer in CDCl_3_ shows the presence of peaks corresponding to both PNIPAM and PEG blocks (Figure 3). The Characteristic resonance of –CH_2_–CH_2_– bond (signal (a)) in PEG units is present in the ^1^H NMR spectrum of the triblock copolymer. The peak (b) at *δ* = 1.09 ppm are ascribed to the methyl group in PNIPAM. The signal at *δ* = 8 ppm (peak ‘d’) is assigned to the proton in the –CH of the PNIPAM.

GPC analysis was performed to determine the molecular weights and molecular weight distributions of the triblock copolymers. Figure 4(a) presents the GPC curves of PNIPAM-b-PEG-b-PNIPAM triblock copolymers.

The GPC analysis showed that the polydispersity index (PDI) is ~1.3, which indicate good control the triblock copolymer during polymerization. This means that the polymerization of triblock copolymer via RAFT is controlled and the number of growing chains remain constant during the reaction. The living nature of the polymerization using the CTA–PEG–CTA RAFT agent is confirmed by the results shown in Figure 4. After the chain extension reaction, the molecular weight increased from 4000 (PEG) to 8400 g/mol (triblock copolymer), indicating that the monomer of NIPAM is successful polymerized by RAFT. The experimental Mn(nmr) values were calculated by ^1^H NMR data according to Equation (1), where *I*_1.09_ ppm, *I*_3.52_ ppm, Mn PEG, M(EG), M(NIPAM) and Mn(CTA–PEG–CTA) represent the integral areas of the signals at 1.09 ppm, 3.52 ppm and the molecular weights of the PEG chain, EG unit, NIPAM unit and CTA–PEG–CTA, respectively.

(1) $$\text{Mn}(\text{nmr})=(I_{1.09}/3)/(I_{3.52}/7)\times \text{Mn}(\text{PEG})/\text{M}(\text{EG})\times \text{M}(\text{NIPAM})+\text{Mn}(\text{CTA}-\text{PEG}-\text{CTA})=7950$$

Table 1 summarizes the characteristics of triblock copolymer and pentablock terpolymers.

**Table 1.** Characterization data of polymers synthesized by RAFT polymerization.

Sample	M_n (NMR)_ (g/mol)	M_n (GPC)_ (g/mol)	PDI	CMC at 60 °C	CMC at pH 4.8	LCST	pH dependant
CTA-PEG-CTA	4000	4000	1.1	–	–	–	–
PNIPAM-b-PEG-b-PNIPAM	7950	8400	1.3	–	–	43	–
PMA-b-PNIPAM-b-PEG-b-PNIPAM-b-PMA	15,700	14,600	1.32	79.4 × 10^−5^ g/mL	23.98 × 10^−6^ g/mL	58	5.8

### Forth step: RAFT polymerization of MAA using PNIPAM-b-PEG-b-PNIPAM as the RAFT agent

PNIPAM-b-PEG-b-PNIPAM was used as the macro-RAFT agent for the subsequent RAFT polymerization of methacrylic acid (MAA) to produce the pentablock terpolymers (Scheme 1). This pentablock copolymer was characterized by FT-IR and ^1^H NMR and GPC.

The FT-IR spectrum of pentablock terpolymer of PMA-b-PNIPAM-b-PEG-b-PNIPAM-b-PMA is shown in Figure 1(e). It is obviously observed in Figure 1(e), that the PMA-b-PNIPAM-b-PEG-b-PNIPAM-b-PMA pentablock terpolymer shows peaks characteristic of PMA, PNIPAM, and PEG. The absorption band at 1730 cm^−1^ is ascribed to the C=O stretching vibrations of the carbonyl group. The strong absorption bands between 960 and 1400 cm^−1^ related to PEG chain. The peak of –NH at 3295 cm^−1^ and a new peak of a carboxyl group (–OH) became visible at 3439 cm^−1^ in the spectrum of the pentablock terpolymer, wholly the C–H stretching bonds are observed at 2912 cm^−1^. All these signals illustrate that the pentablock terpolymer is prepared properly.

Figure 3(b) shows the ^1^H NMR spectrum of PMA-b-PNIPAM-b-PEG-b-PNIPAM-b-PMA pentablock terpolymer. The peaks at *δ* = 1.3 ppm (peaks ‘b, c’) attributed to the protons methyl, *δ* = 1.8–2 ppm (peak ‘e, f’) attributed to the protons methylene (–CH_2_) and *δ* = 11.3 ppm (peak ‘h’) were of –COOH in PMA block. The peaks at *δ* = 3.8 ppm (peak ‘a’) are assigned to the proton in the methylene of the PEG chain and the peak at *δ* = 8 ppm (peak ‘g’) is assigned to the proton in the –NH of the PNIPAM. The appearance of new peaks, indicating that PMA-b-PNIPAM-b-PEG-b-PNIPAM-b-PMA pentablock terpolymer is successfully synthesized.

These results showed that PNIPAM block and PMA block were connected to CTA–PEG–CTA blocks by RAFT polymerization of N-isopropylacrylamid and metacrylic acid monomers. GPC also confirms the synthesis of PMA-b-PNIPAM-b-PEG-b-PNIPAM-b-PMA pentablock terpolymer. When Figure 4(a) is compared with Figure 4(b), the molecular weight increases without a significant change in polydispersity index (PDI = 1.32) as the pentablock terpolymer of PMA-b-PNIPAM-b-PEG-b-PNIPAM-b-PMA is formed, indicating that the pentablock terpolymerization proceeds in a controlled manner. The molecular weight of pentablock terpolymer increased from 8400 (triblock copolymer) to 14,600 g/mol (pentablock terpolymer), indicating that the monomer of MAA is successfully polymerized by RAFT. Also the Mn(nmr) of the pentablock terpolymer is calculated 15,700 g/mol by Equation (1).

### Self-assembly of pentablock terpolymer and CMC determination

Formation of the core-shell micelles of PMA-b-PNIPAM-b-PEG-b-PNIPAM-b-PMA induced by temperature and pH was also studied using pyrene as a fluorescence probe technique. Pyrene has a very small absorption in water and increases when it is transferred into a hydrophobic environment. The intensity ratios of pyrene (*I*_338_/*I*_334_) are used to determine the CMC value of the pentablock terpolymers. Figure 5(a) shows the plot of fluorescence intensity as a function of the concentration of PMA-b-PNIPAM-b-PEG-b-PNIPAM-b-PMA copolymer at 60 °C. The fluorescence intensity is constant at low concentration and increases dramatically above a certain concentration conﬁrming the formation of micelles. The CMC of PMA-b-PNIPAM-b-PEG-b-PNIPAM-b-PMA at 60 °C is about 79.4 × 10^−5^ g/mL determined according to Figure 5(a). The CMC value is one of the important features for polymeric micelles as a drug delivery carrier. The low CMC value is desired to avoid the separation of micelles during the dilution of drug delivery systems by body fluid. With the increase of the polymer concentration from 10^−5^ to 10 g/mL, an abrupt increase in the plot is clearly observed, indicating that the pyrene transfers from water phase into the hydrophobic core of the micelles due to its hydrophobicity and the micro environment of pyrene changes from high-polar water to less-polar micelle. It suggests that the CMC value declines with increasing the compositional ratios of hydrophilic PEG and PMA chains to PNIPAM chains [58].

Figure 5(b) shows the effect of the concentration of PMA-b-PNIPAM-b-PEG-b-PNIPAM-b-PMA on the intensity ratio (*I*_338_/*I*_334_) in acidic aqueous solutions. In an acidic environment (pH 4.8), the *I*_338_/*I*_334_ ratios almost remained constant, ranging between −7 and −5 logC for pentablock terpolymer, which indicated the pyrene probes were located in a hydrophilic environment. But in logC = 4.7 g/mL the *I*_338_/*I*_334_ ratios increase rapidly with an inflection point appeared in the curve, which can be attributed to the CMC of the pentablock terpolymer with pH 4.8. Thus, the CMC of PMA-b-PNIPAM-b-PEG-b-PNIPAM-b-PMA were calculated to be 23.98 × 10^−6^ g/mL. It suggests the formation of a more hydrophobic core due to the presence of more PMA chains in polymer PMA-b-PNIPAM-b-PEG-b-PNIPAM-b-PMA. In acidic solutions with pH 4.8 (<5.5), PMA blocks, which has pKa of about 5.6, can form intra-and/or intermolecular hydrophobic interactions due to protonation of polycarboxylate anions of PMA [59]. In particular, a complexation between carboxylic groups and ethylene oxide repeat units may play an important role in the micelle creation. It is understood that the interpolymer complexation is the non-covalent association between groups on diverse polymer chains, which arrangements because of thermo dynamical compatibility of polymers based on vanderwaals interactions, polyelectrolyte association and hydrogen bonding [60]. In the case of PMA with electron deficient groups and PEG containing regions of high electron density, inter polymer complexes or the complexation are suitable to form due to the collapse of macromolecular chains induced by the PMA chains contracting and the enhanced association between PMA and PEG chains based on the formation of hydrogen bonds. The PMA complexes are more stable than PAA complexesby reason of hydrophobic stabilization of the hydrogen bonds by the *α*-methyl group [61].

### Thermo- and pH-responsive micellization of PMA-b-PNIPAM-b-PEG-b-PNIPAM-b-PMA

**Scheme 2.**
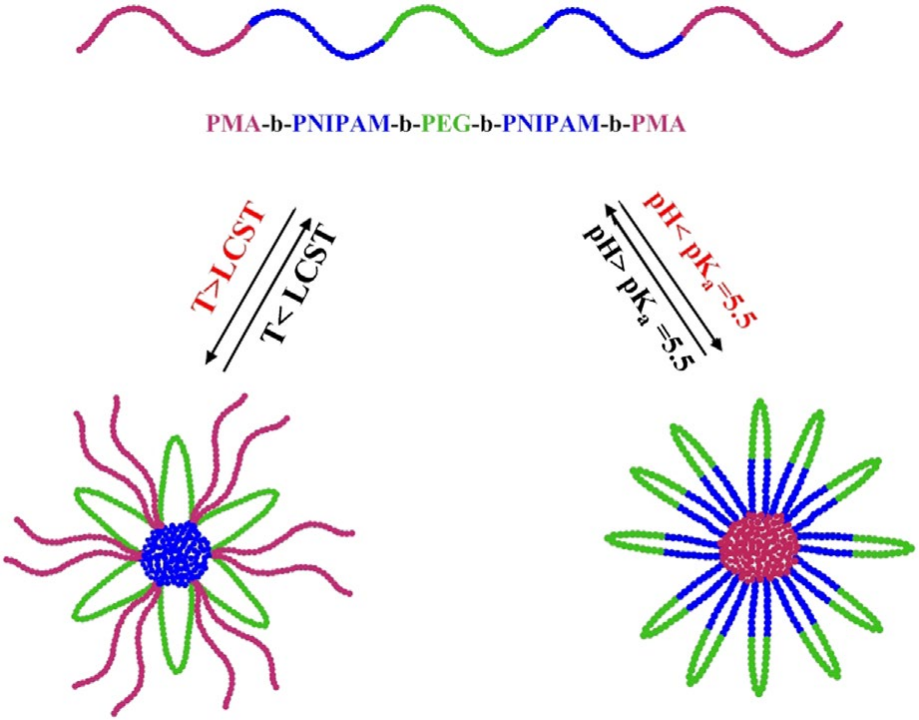
Schematic illustration of the dually-responsive self-assembly behavior of the PMA-b-PNIPAM-b-PEG-b-PNIPAM-b-PMA pentablock copolymer.

The obtained pentablock terpolymer contains a permanently hydrophilic PEG block, a thermo responsive PNIPAM block, and a pH-responsive PMA block. In this study, the PEG block can act as a steric stabilizer for terpolymers, permitting the creation of stable micellar aggregates at middle pH and temperatures. Therefore, for the synthesized pentablock terpolymer, it can be predictable that the terpolymer may show pH- and thermo-responsive micellization behavior through fine tuning of solution pH and temperature (Scheme 2).

Figure 6 illustrates *I*_338_/*I*_334_ intensities, transmittance changes of the terpolymer at wavelength of 495 nm and photographic images with pH changes. In Figure 6, it can be seen that the PMA-b-PNIPAM-b-PEG-b-PNIPAM-b-PMA pentablock terpolymer display a sharp transition behavior at pH ~5.8, which is very near to the pKa (about 5.6) of PMA blocks. At pH < pKa, non ionized PMA adjudge a hyper coiled conformation, and the terpolymer is suitable to form the micelle aggregates due to the intramolecular hydrogen bonding of the COOH in PMA blocks [62]. This behavior is correlated with the pKa ~5.6 of PMA blocks. In the near of pKa, the pentablock terpolymers start to structural transition from large aggregates to self-assembled nanomicelles. When the pH is increased above 7, PMA-b-PNIPAM-b-PEG-b-PNIPAM-b-PMA chains are in stretched conformation due to the electrostatic repulsion between the deprotonated PMA segments, and the solution becomes transparent, and the nanomicelles are separated into unimers [63]. Therefore, the same conclusion that the pentablock terpolymer micelles exhibit pH-response can be inferred from the decrease in the *I*_338_/*I*_334_ intensities (Figure 6(a)) and the increase in the transmittance (Figure 6(b)). However, at pH 7, the micelle cannot be formed because of gradual ionization of –COOH groups in PMA blocks, which cause micelles instability and dissociation of the core–shell structure. This is significant for releasing the enclosed drug molecules [64–66]. Also, Figure 6(c) shows the typical photo images of PMA-b-PNIPAM-b-PEG-b-PNIPAM-b-PMA pentablock terpolymer prepared in the aqueous solution with pH ~7, 4.2 and 2.7. Decreasingof pH to 2 leads to deprotonation and the hydrophobic collapse of the PMA block, indicating the formation of core–shell–corona micelles with the hydrophobic PMA block as the core, the thermo responsive PNIPAM block as the shell and the hydrophilic PEG block as the corona. Actually, the pH has an obvious effect on the micellization behavior of the pentablock terpolymer. Typically, the identification of the core layer, shell layer, and corona layer of core–shell–corona micelles is very hard or difficult.

An ideal LCST would be higher than normal human body temperature so that localized increases in temperature could induce the transformation of the micelles to trigger the release of trapped drug molecules. In the present study, the transmittance of PMA-b-PNIPAM-b-PEG-b-PNIPAM-b-PMA aqueous solutions (1 mg mL^−1^) at pH 7 was measured in the temperature range of 25–80 °C. The LCST was defined as the temperature at which decrease in the transmittance of the aqueous solution of the sample could be considered.

The temperature dependence of transmittance and the resulting LCST of the pentablock terpolymers are displayed in Figure 7. Below the LCST, the PNIPAM block is hydrophilic and the solution is transparent, whereas, above the LCST, the stretched PNIPAM chains collapse to form the cores of the micelles. The PNIPAM chains collapse to form a nanoparticle core while the PEG and PMA chains remain soluble and form a shell to stabilize the micelles, as shown in Scheme 2. As can be seen in Figure 7(a), the critical aggregation temperature of PMA-b-PNIPAM-b-PEG-b-PNIPAM-b-PMA is 58 °C, which is much larger than the homo-PNIPAM (~32 °C). This result is attributed to the presence of hydrophilic polymetacrylic acid and polyethylene glycol blocks in pentablock terpolymer because metacrylic acid and PEG is intrinsically more hydrophilic than NIPAM [38].

### Morphology of pentablock terpolymer

Morphological studies of PMA-b-PNIPAM-b-PEG-b-PNIPAM-b-PMA pentablock terpolymer micelles were done by TEM and SEM, as displayed in Figure 8. The TEM observations reveal that well-deﬁned spherical micelle aggregatesthe structure of the core/shell by self-assembly of pentablock terpolymer in an aqueous solution of pH 5. The TEM size is dependent on environmental pH, with the range of 25–70 nm (Figure 8(a)). In this case, PMA blocks form cores of themicelle. It was noted that the nanoparticles are of a sphere shape with a loose core-shell structure at temperatures below LCST and pH 5.

Also, the surface morphology and particle size of the pentablock terpolymer was observed by SEM. As seen in Figure 8(b), the PMA-b-PNIPAM-b-PEG-b-PNIPAM-b-PMA pentablock ter polymer exhibits the nano and irregularly structured morphology with an average diameter of 70–210 nm in SEM image. The size differences in TEM and SEM image may be due to the agglomerate of the polymer chains in the solid state. This morphology may be originated from the controlled growth of the PNIPAM and PMA from CTA–PEG–CTA macro-RAFT agent.

## Conclusion

In summary, we synthesized PMA-b-PNIPAM-b-PEG-b-PNIPAM-b-PMA dual responsive hydrophilic pentablock terpolymer by two steps of RAFT polymerization for the ﬁrst time. This pentablock terpolymer was containing a pH-responsive PMA block and thermo-responsive PNIPAM blocks, and PEG-based benzoate-type of RAFT agent. The pentablock terpolymer was characterized by ^1^H NMR, FT-IR, GPC, SEM, and TEM. GPC revealed the successful synthesis of target pentablock terpolymer with well controlled molecular weight. The pentablok terpolymers presented the micellization properties with pH- and thermo-responsive behaviors. Fluorescence probe technique and UV–vis analysis conﬁrmed that the obtained pentablock terpolymers self-assembled into core–shell–corona micelles with the pH-responsive PMA block as the core, the thermo responsive PNIPAM block as the shell and the hydrophilic PEG block as the corona, in acidic solution (pH 5.8) and room temperature, while the pentablock terpolymers self-assembled into PNIPAM-core micelles with mixed hydrophilic PEG and pH-responsive PMA coronas at high temperatures (*T* = 58 °C). The resulting nanoparticles had the obvious thermo- and pH-sensitivity. The thermo and pH-responsive micelle aggregates nanoscale spherical shape, with CMC ~79.4 × 10^−5^ g/mL at 60 °C and CMC ~23.98 × 10^−6^ g/mL at pH 4.8.TEM observation revealed that the pentablock terpolymer micelles are nearly spherical shapes in acidic aqueous solution (pH 5) with an average particle size range from 25 to 70 nm. The dual-model functions at normal physiological temperature and pH could have signiﬁcant benefits in the biomedical ﬁeld in the future. These features of the thermo- and pH-responsive micelles open up new chances in the field of smart drug delivery systems.

## Disclosure statement

No potential conflict of interest was reported by the authors.

## References

[1] Li Z, Qiu L, Chen Q, et al pH-sensitive nanoparticles of poly (L-histidine)–poly (lactide-co-glycolide)–tocopheryl polyethylene glycol succinate for anti-tumor drug delivery. Acta Biomater. 2015;11:137–150. 10.1016/j.actbio.2014.09.014 25242647

[2] Zhao T, Guan X, Tang W, et al Preparation of temperature sensitive molecularly imprinted polymer for solid-phase microextraction coatings on stainless steel fiber to measure ofloxacin. Anal Chim Acta. 2015;853:668–675. 10.1016/j.aca.2014.10.019 25467516

[3] Su Y, Hu Y, Du Y, et al Redox-responsive polymer-drug conjugates based on doxorubicin and chitosan oligosaccharide-g-stearic acid for cancer therapy. Mol Pharm. 2015;12(4):1193–1202. 10.1021/mp500710x 25751168

[4] Zhang C, Pan D, Luo K, et al Dendrimer–doxorubicin conjugate as enzyme-sensitive and polymeric nanoscale drug delivery vehicle for ovarian cancer therapy. Polym Chem. 2014;5(18):5227–5235. 10.1039/C4PY00601A

[5] Fu L, Sun C, Yan L Galactose targeted pH-responsive copolymer conjugated with near infrared fluorescence probe for imaging of intelligent drug delivery. ACS Appl Mater Interfaces. 2015;7(3):2104–2115. 10.1021/am508291k 25569169

[6] Shen S, Wang S, Zheng R, et al Magnetic nanoparticle clusters for photothermal therapy with near-infrared irradiation. Biomaterials. 2015;39:67–74. 10.1016/j.biomaterials.2014.10.064 25477173

[7] Liu H, Lin S, Feng Y, et al CO_2_-responsive polymer materials. Polym Chem. 2017;8(1):12–23. 10.1039/C6PY01101B

[8] Lin S, Schattling P, Theato P Thermo- and CO_2_-responsive linear polymers and hydrogels as CO_2_ capturing materials. Sci Adv Mat. 2015;7(5):948–955. 10.1166/sam.2015.2161

[9] Lin S, Theato P CO_2_-Responsive Polymers. Macromol Rapid Commun. 2013;34(14):1118–1133. 10.1002/marc.201300288 23723041

[10] Hong L, Zhang Z, Zhang Y, et al Synthesis and self-assembly of stimuli-responsive amphiphilic block copolymers based on polyhedral oligomeric silsesquioxane. J Polym Sci A Polym Chem. 2014;52(18):2669–2683. 10.1002/pola.v52.18

[11] Zhou Y-N, Zhang Q, Luo Z-H A light and pH dual-stimuli-responsive block copolymer synthesized by copper (0)-mediated living radical polymerization: solvatochromic, isomerization, and ‘schizophrenic’ behaviors. Langmuir. 2014;30(6):1489–1499. 10.1021/la402948s 24472031

[12] Khimani M, Yusa S, Nagae A, et al Self-assembly of multi-responsive poly (N-isopropylacrylamide)-b-poly (N, N-dimethylaminopropylacrylamide) in aqueous media. Eur Polym J. 2015;69:96–109. 10.1016/j.eurpolymj.2015.05.027

[13] Stuart MAC, Huck WT, Genzer J, et al Emerging applications of stimuli-responsive polymer materials. Nat Mater. 2010;9(2):101–113. 10.1038/nmat2614 20094081

[14] Dan M, Huo F, Xiao X, et al Temperature-sensitive nanoparticle-to-vesicle transition of ABC triblock copolymer corona–shell–core nanoparticles synthesized by seeded dispersion RAFT polymerization. Macromolecules. 2014;47(4):1360–1370. 10.1021/ma402370j

[15] Zhou C, Hillmyer MA, Lodge TP Micellization and micellar aggregation of poly (ethylene-alt-propylene)-b-poly (ethylene oxide)-b-poly (N-isopropylacrylamide) triblock terpolymers in water. Macromolecules. 2011;44(6):1635–1641. 10.1021/ma102786q

[16] Strandman S, Zhu X Thermo-responsive block copolymers with multiple phase transition temperatures in aqueous solutions. Prog Polym Sci. 2015;42:154–176. 10.1016/j.progpolymsci.2014.10.008

[17] Jiang X, Lu G, Feng C, et al Poly (acrylic acid)-graft-poly (N-vinylcaprolactam): a novel pH and thermo dual-stimuli responsive system. Polym Chem. 2013;4(13):3876–3884. 10.1039/c3py00415e

[18] Li S, Liu X Synthesis, characterization, and evaluation of enzymatically degradable poly (N-isopropylacrylamide-co-acrylic acid) hydrogels for colon-specific drug delivery. Polym Adv Technol. 2008;19(11):1536–1542.

[19] Chiang W-H, Hsu Y-H, Tang F-F, et al Temperature/pH-induced morphological regulations of shell cross-linked graft copolymer assemblies. Polymer. 2010;51(26):6248–6257. 10.1016/j.polymer.2010.10.038

[20] Karjalainen E, Chenna N, Laurinmäki P, et al Diblock copolymers consisting of a polymerized ionic liquid and poly (N-isopropylacrylamide). Effects of PNIPAM block length and counter ion on self-assembling and thermal properties. Polym Chem. 2013;4(4):1014–1024. 10.1039/C2PY20815F

[21] Li G, Shi L, An Y, et al Double-responsive core–shell–corona micelles from self-assembly of diblock copolymer of poly (t-butyl acrylate-co-acrylic acid)-b-poly (N-isopropylacrylamide). Polymer. 2006;47(13):4581–4587. 10.1016/j.polymer.2006.04.041

[22] Ge Z, Luo S, Liu S Syntheses and self-assembly of poly (benzyl ether)-b-poly (N-isopropylacrylamide) dendritic–linear diblock copolymers. J Polym Sci A Polym Chem. 2006;44(4):1357–1371. 10.1002/(ISSN)1099-0518

[23] Jiang X, Xiong DA, An Y, et al Thermoresponsive hydrogel of poly (glycidyl methacrylate-co-N-isopropylacrylamide) as a nanoreactor of gold nanoparticles. J Polym Sci A Polym Chem. 2007;45(13):2812–2819.

[24] Hou L, Wu P LCST transition of PNIPAM-b-PVCL in water: cooperative aggregation of two distinct thermally responsive segments. Soft Matter. 2014;10(20):3578–3586. 10.1039/c4sm00282b 24664149

[25] Li S, Su Y, Dan M, et al Thermo-responsive ABA triblock copolymer of PVEA-b-PNIPAM-b-PVEA showing solvent-tunable LCST in a methanol–water mixture. Polym Chem. 2014;5(4):1219–1228. 10.1039/C3PY01219K

[26] Chaw C-S, Chooi K-W, Liu X-M, et al Thermally responsive core-shell nanoparticles self-assembled from cholesteryl end-capped and grafted polyacrylamides: drug incorporation and in vitro release. Biomaterials. 2004;25(18):4297–4308. 10.1016/j.biomaterials.2003.10.095 15046920

[27] Pamies R, Zhu K, Kjøniksen A-L, et al Thermal response of low molecular weight poly-(N-isopropylacrylamide) polymers in aqueous solution. Polym Bull. 2009;62(4):487–502. 10.1007/s00289-008-0029-4

[28] Chang K, Rubright NC, Lowery PD, et al Structural optimization of highly branched thermally responsive polymers as a means of controlling transition temperature. J Polym Sci A Polym Chem. 2013;51(9):2068–2078. 10.1002/pola.26596

[29] Bauri K, Roy SG, Arora S, et al Thermal degradation kinetics of thermoresponsive poly (N-isopropylacrylamide-co-N, N-dimethylacrylamide) copolymers prepared via RAFT polymerization. J Therm Anal Calorim. 2013;111(1):753–761. 10.1007/s10973-012-2344-0

[30] Gandhi A, Paul A, Sen SO, et al Studies on thermoresponsive polymers: phase behaviour, drug delivery and biomedical applications. Asian J Pharm Sci. 2015;10(2):99–107. 10.1016/j.ajps.2014.08.010

[31] Li S, Liu X Synthesis, characterization and evaluation of semi-IPN hydrogels consisted of poly (methacrylic acid) and guar gum for colon-specific drug delivery. Polym Adv Technol. 2008;19(5):371–376. 10.1002/(ISSN)1099-1581

[32] Li S, Wang H, Huang W, et al Facile preparation of pH-sensitive poly (acrylic acid-co-acrylamide)/SiO_2_ hybrid hydrogels with high strength by in situ frontal polymerization. Colloid Polym Sci. 2014;292(1):107–113. 10.1007/s00396-013-3050-6

[33] Li S, Yan S Rapid synthesis of macroporous graphene oxide/poly (acrylic acid-co-acrylamide) nanocomposite hydrogels with pH-sensitive behavior by frontal polymerization. RSC Adv. 2016;6(40):33426–33432. 10.1039/C6RA03214A

[34] Li S, Yang Y, Li H, et al pH‐responsive semi‐interpenetrating networks hydrogels of poly (acrylic acid‐acrylamide‐methacrylate) and amylose. I. Synthesis and characterization. J Appl Polym Sci. 2007;106(6):3792-3799. 10.1002/app.v106:6

[35] Li S, Yang Y, Yang X, et al In vitro degradation and protein release of semi-IPN hydrogels consisted of poly (acrylic acid-acrylamide-methacrylate) and amylose. J Appl Polym Sci. 2007;105(6):3432–3438. 10.1002/(ISSN)1097-4628

[36] Zeinali E, Haddadi-Asl V, Roghani-Mamaqani H Nanocrystalline cellulose grafted random copolymers of N-isopropylacrylamide and acrylic acid synthesized by RAFT polymerization: effect of different acrylic acid contents on LCST behavior. RSC Adv. 2014;4(59):31428–31442. 10.1039/C4RA05442C

[37] Gao X, Cao Y, Song X, Zhang Z, Xiao C, He C, et al pH- and thermo-responsive poly (N-isopropylacrylamide-co-acrylic acid derivative) copolymers and hydrogels with LCST dependent on pH and alkyl side groups. J Mater Chem B. 2013;1(41):5578–5587. 10.1039/c3tb20901f 32261182

[38] Yin X, Hoffman AS, Stayton PS Poly (N-isopropylacrylamide-co-propylacrylic acid) copolymers that respond sharply to temperature and pH. Biomacromolecules. 2006;7(5):1381–1385. 10.1021/bm0507812 16677016

[39] Li G, Song S, Guo L, et al Self-assembly of thermo- and pH-responsive poly (acrylic acid)-b-poly (N-isopropylacrylamide) micelles for drug delivery. J Polym Sci A Polym Chem. 2008;46(15):5028–5035. 10.1002/(ISSN)1099-0518

[40] Bastakoti BP, Guragain S, Nakashima K, et al Stimuli-induced core-corona inversion of micelle of poly (acrylic acid)-block-poly (n-isopropylacrylamide) and its application in drug delivery. Macromol Chem Phys. 2015;216(3):287–291. 10.1002/macp.201400440

[41] Guragain S, Bastakoti BP, Nakashima K Schizophrenic micellization of poly(ethylene oxide-b-methacrylic acid) induced by phosphate and calcium ions. J. Colloid Interface Sci. 2010;350(1):63–68. 10.1016/j.jcis.2010.06.007 20580372

[42] Wang D, Wu T, Wan X, et al Purely salt-responsive micelle formation and inversion based on a novel schizophrenic sulfobetaine block copolymer: structure and kinetics of micellization. Langmuir. 2007;23(23):11866–11874. 10.1021/la702029a 17929848

[43] Liu S, Armes SP Synthesis and aqueous solution behavior of a pH-responsive schizophrenic diblock copolymer. Langmuir. 2003;19(10):4432–4438. 10.1021/la020951l

[44] Schilli CM, Zhang M, Rizzardo E, et al A new double-responsive block copolymer synthesized via raft polymerization: poly(N-isopropylacrylamide)-block-poly(acrylic acid). Macromolecules. 2004;37(21):7861–7866. 10.1021/ma035838w

[45] Akbarzadeh A, Samiei M, Joo SW, et al Synthesis, characterization and *in vitro* studies of doxorubicin-loaded magnetic nanoparticles grafted to smart copolymers on A549 lung cancer cell line. J nanobiotechnol. 2012;10(1):1. 10.1186/1477-3155-10-46 PMC3605180 23244711

[46] Alves TVG, Tavares EJM, Aouada FA, et al Thermal analysis characterization of PAAm-co-MC hydrogels. J Therm Anal Calorim. 2011;106(3):717–724. 10.1007/s10973-011-1572-z

[47] Zhang H Controlled/‘living’ radical precipitation polymerization: a versatile polymerization technique for advanced functional polymers. Eur Polym J. 2013;49(3):579–600. 10.1016/j.eurpolymj.2012.12.016

[48] Abbasian M, Mahmoodzadeh F Synthesis of antibacterial silver–chitosan-modified bionanocomposites by RAFT polymerization and chemical reduction methods. J Elastomers Plast. 2016;0095244316644858.

[49] Abbasian M, Masoumi B, Rashidzadeh B Versatile method via reversible addition-fragmentation transfer polymerization for synthesis of poly styrene/ZnO–nanocomposite. Polym Eng Sci. 2016;56(2):187–195. 10.1002/pen.v56.2

[50] Abbasian M, Ahmadkhani L Synthesis of conductive PSt-g-PANi/TiO_2_ nanocomposites by metal catalyzed and chemical oxidative polymerization. Des Monomers Polym. 2016;19(7):585–595. 10.1080/15685551.2016.1187435

[51] Abbasian M, Aali NK, Shoja SE Synthesis of poly (methyl methacrylate)/zinc oxide nanocomposite with core-shell morphology by atom transfer radical polymerization. J Macromol Sci A. 2013;50(9):966–975. 10.1080/10601325.2013.813814

[52] Abbasian M, Massomi B, Rashidzadeh B, et al Versatile method for synthesis of electrically conductive polypyrrole-polystyrene clay nanocomposites using ATRP and chemical polymerisation methods. J Exp Nanosci. 2015;10(11):844–858. 10.1080/17458080.2014.910617

[53] Karaj-Abad SG, Abbasian M, Jaymand M Grafting of poly [(methyl methacrylate)-block-styrene] onto cellulose via nitroxide-mediated polymerization, and its polymer/clay nanocomposite. Carbohyd Polym. 2016;152:297–305. 10.1016/j.carbpol.2016.07.017 27516276

[54] Smith AE, Xu X, McCormick CL Stimuli-responsive amphiphilic (co)polymers via RAFT polymerization. Prog Polym Sci. 2010;35(1):45–93. 10.1016/j.progpolymsci.2009.11.005

[55] Chiefari J, Mayadunne RT, Moad CL, et al Thiocarbonylthio compounds (SC (Z) SR) in free radical polymerization with reversible addition-fragmentation chain transfer (RAFT polymerization). Effect of the activating group Z. Macromolecules. 2003;36(7):2273–2283. 10.1021/ma020883+

[56] Lowe AB, McCormick CL Reversible addition–fragmentation chain transfer (RAFT) radical polymerization and the synthesis of water-soluble (co)polymers under homogeneous conditions in organic and aqueous media. Prog Polym Sci. 2007;32(3):283–351. 10.1016/j.progpolymsci.2006.11.003

[57] Le T, Moad G, Rizzardo E, et al., editors. PCT Int. Appl. WO 9801478 A1 980115 Chem Abstr; 1998.

[58] Luo Y-L, Huang R-J, Zhang L-L, et al Dual-responsive polyacrylate copolymer micelles with PMAA and PNIPAAm graft brushes: physicochemical properties and prednisone release. Colloids Surf A Physiochem Eng Asp. 2013;436:1175–1185. 10.1016/j.colsurfa.2013.08.018

[59] Guo M, Yan Y, Zhang H, et al Magnetic and pH-responsive nanocarriers with multilayer core–shell architecture for anticancer drug delivery. J Mater Chem. 2008;18(42):5104–5112. 10.1039/b810061f

[60] Lowman A, Peppas N Molecular analysis of interpolymer complexation in graft copolymer networks. Polymer. 2000;41(1):73–80. 10.1016/S0032-3861(99)00159-7

[61] Luo Y-L, Yu W, Xu F pH-responsive PMAA-b-PEG-b-PMAA triblock copolymer micelles for prednisone drug release and release kinetics. Polym Bull. 2012;69(5):597–620. 10.1007/s00289-012-0774-2

[62] Chao G, Deng H, Huang Q, et al Preparation and characterization of pH sensitive semi-interpenetrating network hydrogel based on methacrylic acid, bovine serum albumin (BSA), and PEG. J Polym Res. 2006;13(5):349–355.

[63] Arimura H, Ohya Y, Ouchi T Formation of core-shell type biodegradable polymeric micelles from amphiphilic poly(aspartic acid)-block-polylactide diblock copolymer. Biomacromolecules. 2005;6(2):720–725. 10.1021/bm0494491 15762635

[64] Soppimath KS, Tan DW, Yang YY pH-triggered thermally responsive polymer core-shell nanoparticles for drug delivery. Adv Mater. 2005;17(3):318–323. 10.1002/(ISSN)1521-4095

[65] Lee ES, Shin HJ, Na K, et al Poly (L-histidine)–PEG block copolymer micelles and pH-induced destabilization. J Controlled Release. 2003;90(3):363–374. 10.1016/S0168-3659(03)00205-0 12880703

[66] Lee ES, Na K, Bae YH Polymeric micelle for tumor pH and folate-mediated targeting. J Controlled Release. 2003;91(1):103–113. 10.1016/S0168-3659(03)00239-6 12932642

