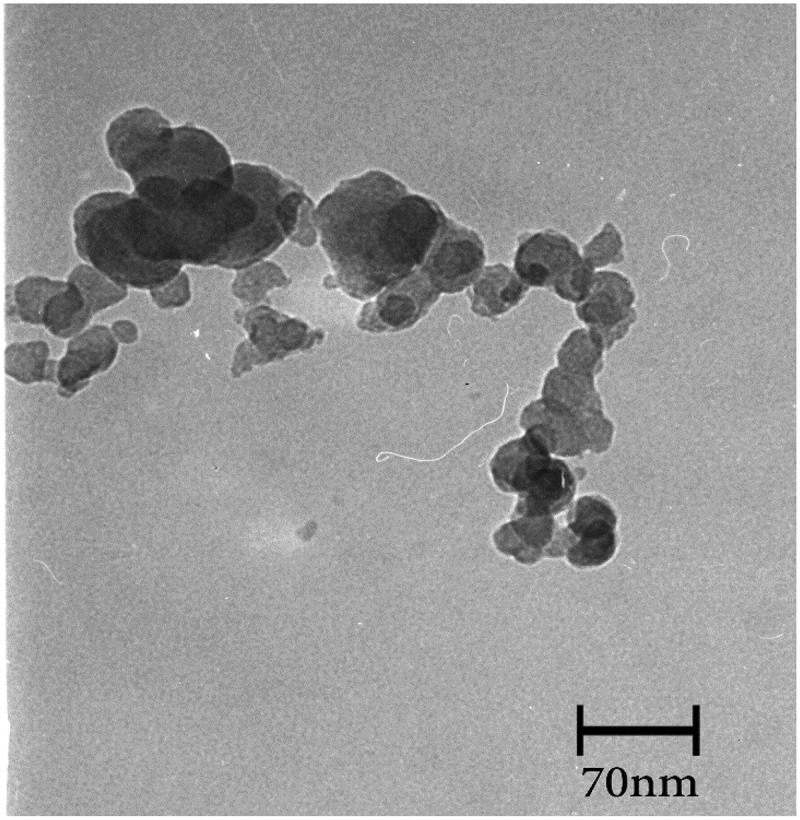# Correction

**DOI:** 10.1080/15685551.2020.1827621

**Published:** 2020-10-07

Article title: Synthesis of sharply thermo and PH responsive PMA-b-PNIPAM-b-PEG-b-PNIPAM-b-PMA by RAFT radical polymerization and its Schizophrenic micellization in aqueous solutions

Authors: Mojtaba Abbasian, Lida Ahmadkhani, and Abolfazl Akbarzadeh

Journal: Designed Monomers and Polymers

Bibliometrics: VOL. 20, NO. 1, 406 – 418

DOI: 10.1080/15685551.2017.1314654

We have been notified by the authors that Figure 8a had been incorrectly updated in the published article. Figure 8a should have appeared as shown below. This correction has not changed the description, interpretation, or the original conclusions of the article.

**Figure:**